# Development of a target concentration intervention to individualize paroxysmal nocturnal hemoglobinuria treatment with pegcetacoplan

**DOI:** 10.1007/s00277-024-05699-8

**Published:** 2024-03-08

**Authors:** Mendy ter Avest, Saskia M.C Langemeijer, Nicole M.A. Blijlevens, Nicole C.A.J. van de Kar, Rob ter Heine

**Affiliations:** 1 Department of Pharmacy, Radboud University Medical Centre, P.O. Box 9101, Nijmegen, 6500 HB The Netherlands; 2 Department of Hematology, Radboud University Medical Centre, Nijmegen, The Netherlands; 3 Department of Pediatric Nephrology, Radboud University Medical Centre, Nijmegen, The Netherlands

**Keywords:** Pegcetacoplan, Paroxysmal nocturnal hemoglobinuria, Pharmacokinetics, Pharmacodynamics, Complement

## Abstract

Pegcetacoplan (Aspaveli®/Empaveli™) is a factor C3 inhibitor that is approved for the treatment of paroxysmal nocturnal hemoglobinuria. An individualized dosing strategy might be useful to improve patient-friendliness and cost-effectiveness of this very expensive drug. Therefore, the aim of this study was to develop an individualized treatment regimen for pegcetacoplan based on the pharmacokinetic-pharmacodynamic data of the manufacturer. We conducted a clinical trial simulation with the approved dosing regimen of 1080 mg twice-weekly and a target concentration intervention-based dosing regimen in patients with and without prior eculizumab use. For eculizumab-naïve patients, the target concentration intervention-based dosing regimen resulted in a comparable fraction of patients with LDH normalization (LDH < 226 U/L) and hemoglobulin normalization (> 12 g/dL) compared to the approved regimen (LDH 50.2% and 50.0% respectively and hemoglobulin 45.6% and 44.4%). A modest dose reduction of ~ 5% was possible with target concentration intervention-based dosing. An intensified dosing interval was necessary in 2.3% of the patients however an interval prolongation was possible in 28.2% of the patients. Similar results were obtained for patients prior treated with eculizumab. In this study we show the potential of an individualized dosing regimen of pegcetacoplan with can improve patient friendliness in approximately 30% of the patients and improve therapy in approximately 2% of the patients at slightly reduced costs.

**Supplementary Information:**

The online version contains supplementary material available at 10.1007/s00277-024-05699-8.

## Introduction

Pegcetacoplan (Aspaveli®/Empaveli™) is the first-in-class complement factor C3 inhibitor that has recently been approved for the treatment of paroxysmal nocturnal hemoglobinuria (PNH) [1]. PNH is a very rare and severe hematological disease characterized by complement-mediated hemolysis, thrombosis and bone marrow failure [2].

By binding to C3, pegcetacoplan inhibits the cleavage of C3 into C3a and C3b. It also binds to C3b, thereby inhibiting the activity of convertases that contain a C3b subunit including C3 and C5 convertases of the alternative pathway and C5 convertase of the classical pathway [1]. As pegcetacoplan targets complement factor C3, it can prevent both intravascular and extravascular hemolysis and was superior to eculizumab in improving hemoglobin and clinical and hematologic outcomes in patients with PNH [3]. Pegcetacoplan is approved for the treatment of adult PNH patients in the United States (EMPAVELI™) [1], for adult PNH patients who are anemic after treatment with a complement factor C5 inhibitor for at least 3 months in Europe (ASPAVELI©) [4] and for adult PNH patients who are anemic after treatment with a C5 inhibitor for at least 3 months or intolerant to C5 inhibitors in Australia (EMPAVELI) [5].

Currently, C5-inhibition with eculizumab or ravulizumab is first choice treatment in PNH patients with hemolytic disease or thromboembolic complications [6]. Since C5 inhibitors only prevent intravascular hemolysis, extravascular hemolysis in the liver and spleen due to opsonization of PNH erythrocytes with C3 fragments, can still occur. This can manifest as persistent anemia and need for blood transfusions, despite treatment with a C5 inhibitor [3].

Although pegcetacoplan administration is associated with reduced health care costs compared to C5 inhibitors eculizumab and ravulizumab [7, 8], therapy is still very expensive with annual costs of US$458.000 per patient [9]. The recommended dose of pegcetacoplan in adults is 1080 mg twice weekly, administered subcutaneously [1]. A clear relationship between pegcetacoplan concentrations in the blood with lactate dehydrogenase (LDH) and hemoglobin response has been described [1]. These data imply that an individualized dosing strategy might be useful to further individualize treatment, to improve patient-friendliness and cost-effectiveness. Therefore, the aim of this study was to develop an individualized treatment regimen for pegcetacoplan based on the pharmacokinetic-pharmacodynamic data of the manufacturer of pegcetacoplan.

## Methods

We conducted a clinical trial simulation with the approved dosing regimen of 1080 mg twice-weekly and a target concentration intervention-based dosing regimen (See Table 1) in adult patients with and without prior eculizumab use. The population pharmacokinetic-pharmacodynamic model that was used for the simulations was almost completely extracted from the drug approval review documents of the Food and Drug Administration (FDA) [1]. All simulations were performed with the software package NONMEM v7.5 (Icon PLC, Dublin, Ireland).

**Table 1:** Target concentration intervention dosing regimen for pegcetacoplan

Start			
*Standard dose* 1080 mg on day 1-4-8-11 of every 14 days			
**Dose adjustment based on TDM**			
*Trough concentration and LDH level [after > 7 weeks]*			
**Concentration (µg/mL)**		**LDH (U/L)**	**Advise**
< 396		> 226	1080 mg on day 1-3-5-8-10-12 of every 14 days
< 296		< 226	Standard dose
396–597			
> 597		> 226	
597–900		< 226	1080 mg on day 1-6-11 of every 14 days
> 900		< 226	1080 mg on day 1–8 of every 14 days
**In case of interval prolongation**			
*LDH level after 4 weeks*			
**LDH**	**Dose**		**Advise**
< 226			Continue current dosing strategy
> 226	1080 mg on day 1-6-11 of every 14 days		Switch to Standard dose
> 226	1080 mg on day 1–8 of every 14 days		Switch to 1080 mg on day 1-6-11 of every 14 days

Pharmacokinetics of pegcetacoplan were described by the manufacturer with a one-compartment disposition model after absorption from the subcutaneous compartment, with first order elimination with baseline body weight as covariate on clearance and volume of distribution. Subcutaneous absorption was described with a one-transit compartment. Clearance and volume of distribution of pegcetacoplan in PNH patients were estimated to be 0.015 L/h and 3.9 L, respectively and obtained from the summary of product characteristics from the European Medicines Agency [4].

The relationship between drug concentrations and hemoglobin level was described by the manufacturer with a sigmoidal direct maximum effect model, with individual observed baseline hemoglobin level and creatinine clearance as covariates on the maximum effect (E_max_). The effect of these covariates on E_max_ was extracted from the reported data for the described effect data for the 5th, 50th and 95th percentiles of individual observed baseline hemoglobin level and creatinine clearance on Emax in the drug approval package of the FDA [1]. Furthermore, the relationship between pegcetacoplan plasma concentrations and LDH levels was also described with a sigmoidal direct Emax model. The model code and a schematic representation of the model have been added to the supplemental material. We evaluated the approved dosing regimen of 1080 mg twice weekly and an individualized dosing regimen. For the individualized dosing regimen, we adjusted the dosing interval after trough level measurement on day 49 of treatment (see Table 1 for proposal), developed by testing various algorithms (data not shown). A pegcetacoplan concentration between 396 µg/mL (EC_90_ for hemoglobin response) and 597 µg/mL (EC_99_ for hemoglobin response) was considered therapeutic [1]. Simulations were based on the demographic data of 1171 representative adult patients, sampled from the NHANES database [3, 10] with the following characteristics: weight (median: 73.8 kg, inter quartile range: 63.6–83.9 kg); Length (1.67 m, 1.60-1.75 m). Sex (female: 49.8%). We predicted a median (IQR) baseline hemoglobin level of 8.6 (7.9–9.2) G/dL and a baseline LDH of 2124 (1682–2682) U/L (in case of eculizumab naïve patients) or 249 (197–314) U/L (prior eculizumab use), based on the drug approval data [1]. For each scenario, we predicted hemoglobin levels, LDH levels and pegcetacoplan trough concentration during therapy and calculated the proportion of patients with hemoglobin levels < 12 g/dL (lower limit of normal for female patients), LDH levels > 226 g/dL (upper limit of normal range) and pegcetacoplan trough concentrations < 396 µg/mL (signs of inadequate therapy) at week 16 of treatment. Furthermore, we calculated the mean annual maintenance dosing costs per patient, assuming costs of US$4,404 per subcutaneous injection (yearly drug costs US$458,000 for 104 injections) [9]. Lastly, for the target concentration intervention dosing regimen we calculated the proportion of patients on an intensified and prolonged dosing regimen and we compared pegcetacoplan concentrations, hemoglobin and LDH concentrations predicted for the alternative dosing strategy and the standard dose for each individual.

## Results

**Fig. 1:**
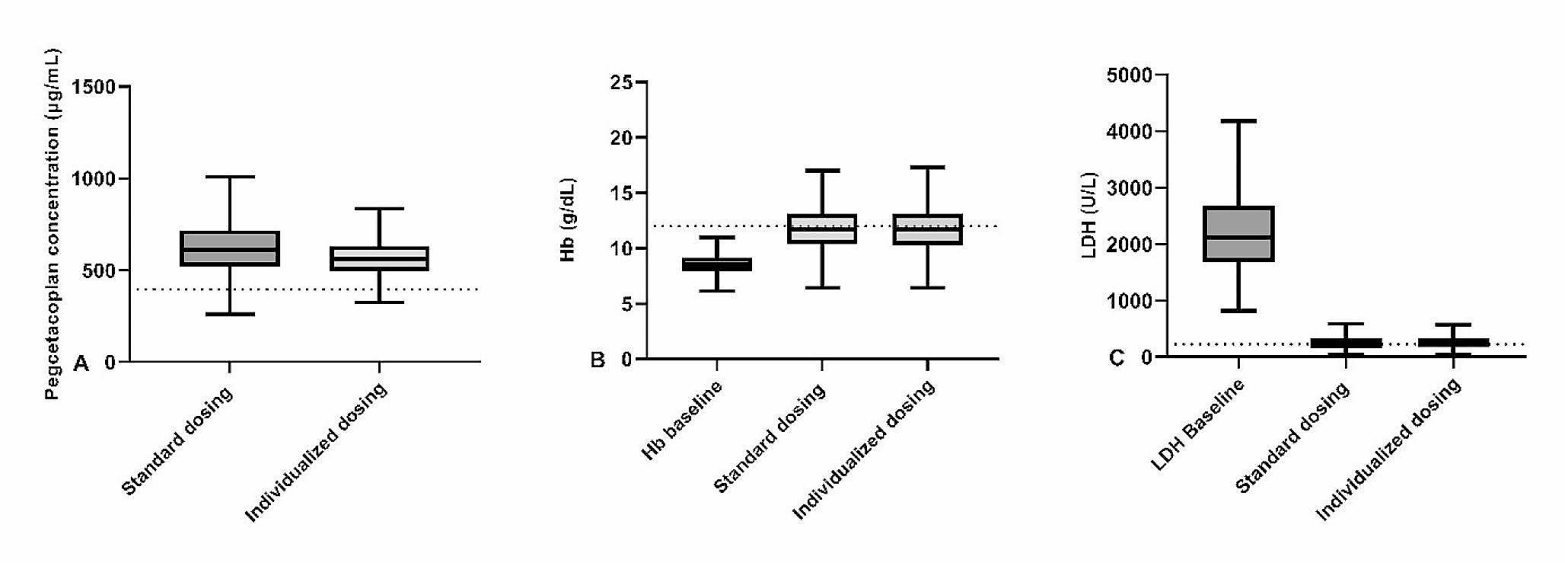
Tukey box-and-whisker plots for pegcetacoplan trough concentrations (**A**), Hemoglobin levels (Hb) (**B**) and lactate dehydrogenase (LDH) (**C**) at week 16 of treatment for both standard dosing and TDM based dosing. The dotted lines represents a pegcetacoplan concentration of 396 µg/ml (EC_90_) (**A**), a hemoglobin level of 12.0 g/dL (lower limit of normal range for female patients) (**B**) and an LDH level of 226 U/L (upper limit of normal range) (**C**)

**Fig. 2:**
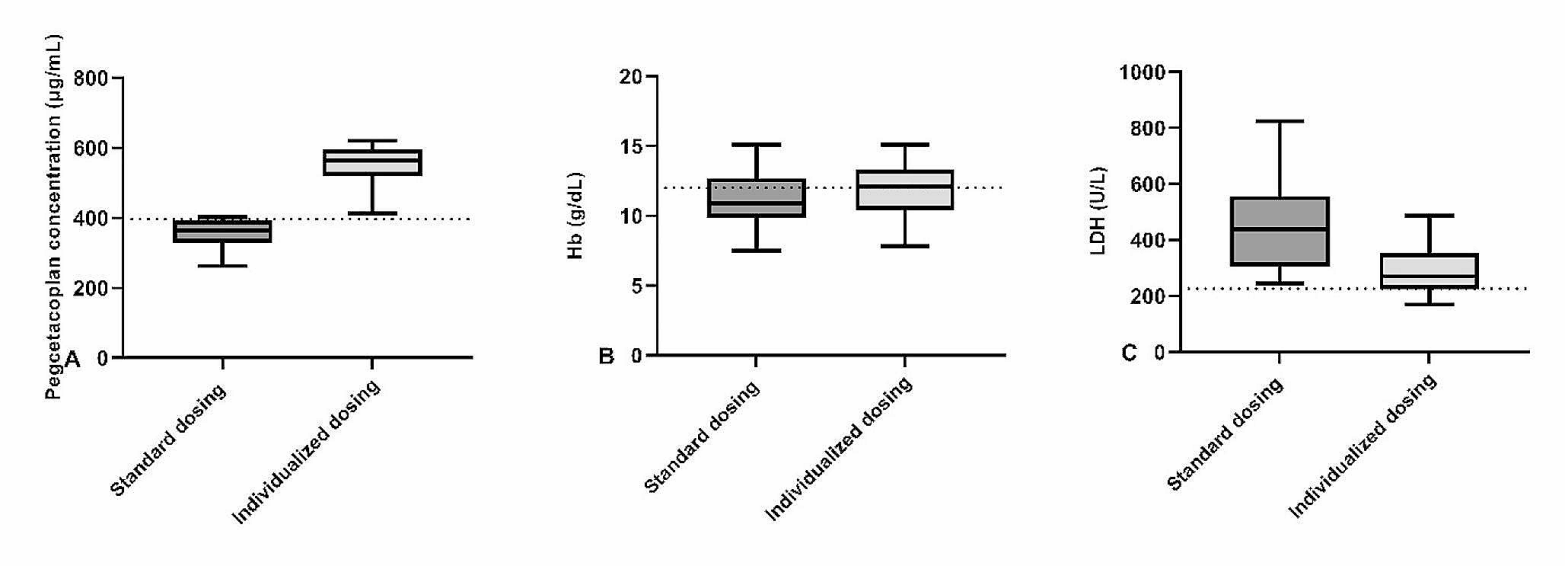
Tukey box-and-whisker plots for pegcetacoplan trough concentrations (**A**), Hemoglobin levels (Hb) (**B**) and lactate dehydrogenase (LDH) (**C**) at week 16 of treatment for both standard dosing and an intensified TDM based dosing strategy for patients with a pegcetacoplan trough concentrations < 396 µg/ml after 7 weeks of treatment. The dotted lines represents a pegcetacoplan concentration of 396 µg/ml (EC_90_) (**A**), a hemoglobin level of 12.0 g/dL (lower limit of normal range for female patients) (**B**) and an LDH level of 226 U/L (upper limit of normal range) (**C**)

**Fig. 3:**
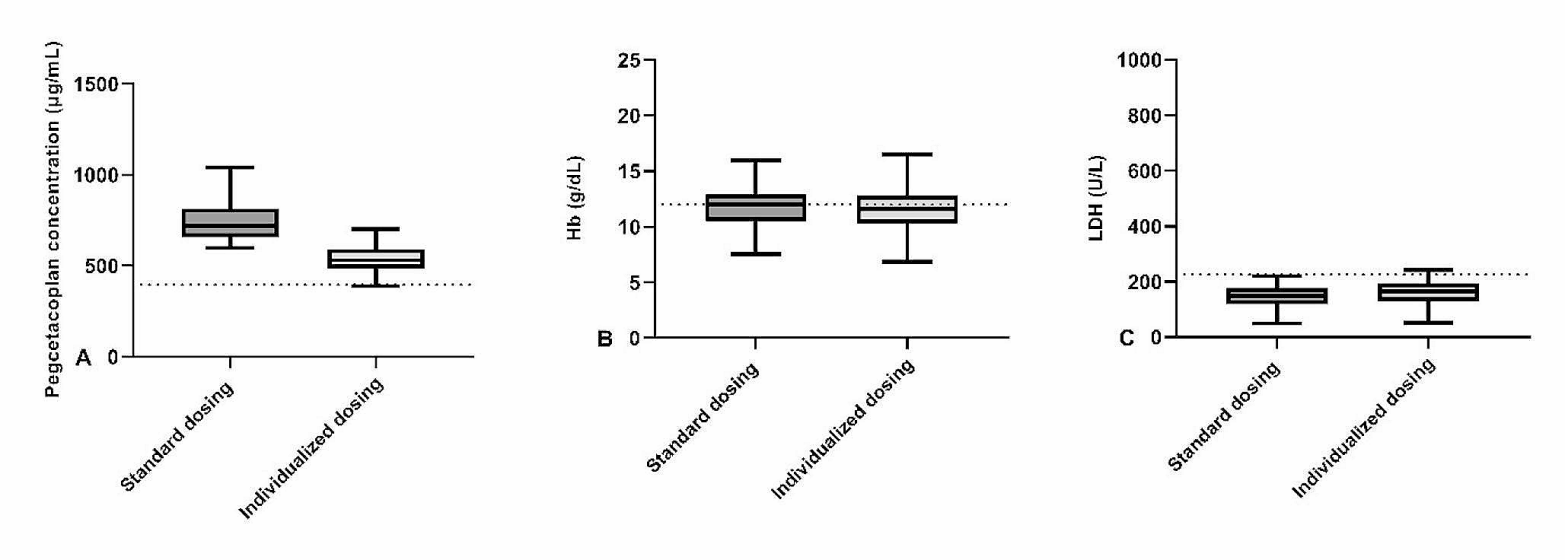
Tukey box-and-whisker plots for pegcetacoplan trough concentrations (**A**), Hemoglobin (Hb) levels (**B**) and lactate dehydrogenase (LDH) (**C**) at week 16 of treatment for both standard dosing and an prolonged TDM based dosing strategy for patients with a pegcetacoplan trough concentrations > 597 µg/ml after 7 weeks of treatment. The dotted lines represents a pegcetacoplan concentration of 396 µg/ml (EC_90_) (**A**), a hemoglobin level of 12.0 g/dL (lower limit of normal range for female patients) (**B**) and an LDH level of 226 U/L (upper limit of normal range) (**C**)

The predicted pegcetacoplan trough concentrations, hemoglobin levels and LDH levels at week 16 of treatment for eculizumab-naïve patients for both the approved dosing regimen and the individualized dosing regimen are depicted in Fig. 1A-C. Pegcetacoplan trough concentrations were highly variable between patients for the approved dosing regimen and this variability could only be moderately reduced with the target concentration intervention. For the standard dosing regimen, 3% of the patients in the standard dosing group exhibited subtherapeutic pegcetacoplan concentrations (< 396 µg/mL) at steady state, compared to 1% of the patients in the target concentration intervention group. Median (IQR) LDH concentrations and hemoglobin levels at steady state were comparable for both the standard dosing regimen (LDH: 225 (159–331) U/L; hemoglobin: 11.7 (10.4–13.1) g/dL and the target concentration intervention dosing regimen (LDH 226 (168–327) U/L; hemoglobin: 11.7 (10.3–13.1) g/dL. The fraction of patients with LDH normalization (LDH < 226 U/L) at week 16 of treatment were 50.2% and 50.0% for the standard dosing regimen and target concentration intervention regimen respectively. The fraction of patients with hemoglobin normalization (hemoglobin > 12 g/dL) at week 16 of treatment were 45.6% and 44.4% respectively. The mean yearly maintenance dose costs per patients for the standard dosing regimen and the target concentration intervention regimen were US$458,000 and US$436,358, respectively, showing a modest potential of ~ 4.7% reduction in yearly drug costs. An intensified dosing interval was necessary in 2.3% of the patients and interval prolongation was possible in 28.2% of the patients. Figures 2A-C and 3A-C show the predicted pegcetacoplan trough concentrations, hemoglobin levels and LDH levels at week 16 of treatment for both the approved dosing regimen and the individualized intensified (Fig. 2) and prolonged (Fig. 3) dosing regimen. For patients with a pegcetacoplan trough concentration of < 396 µg/mL and LDH > 226 U/L after 7 weeks of treatment, standard dosing will result in median (IQR) pegcetacoplan concentrations of 364.3 (331.9-390.1) µg/mL, hemoglobin levels of 10.9 (10.1–12.4) g/dL and LDH levels of 439 (307–554) U/L at week 16 of treatment. When an intensified dosing regimen of thrice weekly pegcetacoplan was used, median (IQR) pegcetacoplan concentrations increased to 564.5 (521.7-596.6) µg/mL, hemoglobin levels increased to 12.0 (10.7–13.2) g/dL and LDH decreased to 270 (226–342) U/L at week 16 of treatment.

For patients with a pegcetacoplan trough concentration of > 597 µg/mL and LDH < 226 U/L after 7 weeks of treatment, standard dosing will result in median (IQR) pegcetacoplan concentrations of 720.7 (656.9-811.5) µg/mL, hemoglobin levels of 12.0 (10.6–12.9) g/dL and LDH levels of 148.9 (119.1-176.9) U/L at week 16 of treatment. When a prolonged dosing regimen was used, median (IQR) pegcetacoplan concentrations decreased to 564.5 (521.7-596.6) µg/mL, hemoglobin levels were 11.6 (10.3–12.8) g/dL and LDH levels were 165 (129–194) U/L at week 16 of treatment.

Similar results were obtained for patients with prior eculizumab use. Differences were likely due to the pharmacokinetic-pharmacodynamic model that was used, in which different formula describe the effect of pegcetacoplan on LDH for patients with and without prior eculizumab use.

Median (IQR) LDH concentrations at steady state were comparable for both the standard dosing regimen (LDH: 170 (124–242) U/L and the TDM based dosing regimen (LDH 174 (125–241) U/L. The fraction of patients with LDH normalization (LDH < 226 U/L) at week 16 of treatment were 70.4% in both groups. The mean yearly maintenance dose costs per patients for the standard dosing regimen and the TDM based dosing regimen were US$458,000 and US$420,626, respectively, showing a potential of ~ 8.2% reduction in yearly drug costs. An intensified dosing interval was necessary in 1.4% of the patients and interval prolongation was possible in 36.7% of the patients. For other results, see supplemental material.

## Discussion

We showed a proof-of-concept for dose individualization of pegcetacoplan in PNH patients that could improve patient-friendliness in ~ 30% of the patients by interval prolongation and could improve therapy outcomes in patients with subtherapeutic pegcetacoplan concentrations (~ 2% of the patients). With an individualized dose, drug costs can be reduced by 4.7% (~ US$21.500 per patient per year) in eculizumab-naïve patients and by 8.1% (~ US$37.000) in patients with prior eculizumab use. The less frequent administration in a subpopulation may also cause a reduction in costs due to lost productivity.

In case of a target concentration dosing strategy, LDH concentrations and hemoglobin levels at steady-state were comparable with the approved dosing regimen, which suggests that pegcetacoplan exposure stayed around the plateau phase of the concentration-effect curve.

For eculizumab-naïve patients with subtherapeutic (< 396 µg/mL) pegcetacoplan concentrations and LDH > 226 U/L we show that median LDH levels could be decreased from 439 (307–554) U/L to 270 (226–342) U/L and hemoglobin levels could be increased from 10.9 (10.1–12.4) g/dL to 12.0 (10.7–13.2) g/dL when therapy is intensified to thrice weekly dosing.

The severity of breakthrough hemolysis seems to be higher with the use of proximal complement inhibitors such as pegcetacoplan than observed with terminal complement inhibitors like eculizumab [11, 12]. Intensifying the dosing interval in patients with subtherapeutic concentrations might prevent breakthrough hemolysis. In the phase 3 trial directly comparing pegcetacoplan to eculizumab, two of the three patients in the pegcetacoplan group that withdrew from the study treatment had lower than average pegcetacoplan concentrations at steady-state [3]. We advise that with prolonging the dosing interval, patients should be closely monitored for signs and symptoms of breakthrough hemolysis.

An intravenous formulation of pegcetacoplan and an intensified subcutaneous regimen are currently under investigation for treatment of acute hemolytic events [12]. Our proposed target concentration intervention regimen might also be of added value in optimizing therapy in these cases.

The use of a target concentration intervention regimen for pegcetacoplan requires the development of a suitable bioanalytical method to measure pegcetacoplan concentrations. A liquid chromatography tandem mass spectrometry (LC-MS/MS) method was developed and validated for the registration studies of the manufacturer [13]. LC-MS/MS is a commonly used for bioanalytical analysis and associated with acceptable costs [14], especially when the analysis are being centered in for example a national laboratory.

We used a modified version of the pharmacokinetic-pharmacodynamic model of the manufacturer, that was described in the drug approval data [1, 4, 5]. In the pharmacokinetic-pharmacodynamic model of the manufacturer, time-varying C3 concentration was a covariate on clearance and volume of distribution, but it was not specified how this covariate impacts these parameters. Therefore, this covariate was not added to our pharmacokinetic-pharmacodynamic model. The manufacturer stated that steady-state pegcetacoplan exposure at the 5th and 95th percentile of C3 level over time were respectively ~ 6% lower and ~ 4% higher [1]. We consider this effect not clinically relevant and expect no relevant impact on our conclusions.

The pharmacokinetic and pharmacokinetic model of the manufacturer is based on data from the clinical studies with pegcetacoplan with restrictive eligibility criteria and might not entirely be illustrative for the real-world population. Therefore, our proposed dosing regimen should be prospectively evaluated in a real-world setting to confirm non-inferiority with hemoglobin normalization, breakthrough hemolysis, LDH normalization, pharmacokinetic target attainment, and treatment costs.

In this study we show the potential of an individualized dosing regimen of pegcetacoplan with can improve patient friendliness in approximately 30% of the patients and improve therapy in approximately 2% of the patients at slightly reduced costs.

### Electronic supplementary material

Below is the link to the electronic supplementary material.

Supplementary Material 1
