# Supplementary material for: Development of a target concentration intervention to individualize paroxysmal nocturnal hemoglobinuria treatment with pegcetacoplan
Source: Ann Hematol. 2024 Mar 8;103(7):2267–72. doi: 10.1007/s00277-024-05699-8 (PMC11224070; doi:10.1007/s00277-024-05699-8)
Supplement: Supplementary file 1 — Supplementary Material 1 [file 277_2024_5699_MOESM1_ESM.docx]

**SUPPLEMENTAL MATERIAL**

**3. Results**

Patients with prior eculizumab use

The predicted pegcetacoplan trough concentrations, hemoglobin levels and LDH levels at week 16 of treatment for eculizumab-naïve patients for both the approved dosing regimen and the individualized dosing regimen are depicted in supplemental Figure S1A-C. A large variability in pegcetacoplan concentration was observed for the approved dosing regimen, which can partly be reduced with an individualized dosing regimen, while maintaining therapeutic exposure. For the standard dosing regimen, 3% of the patients in the standard dosing group exhibited subtherapeutic pegcetacoplan concentrations (<396 µg/mL) at steady state, compared to 2% of the patients in the individualized dosing group. Median (IQR) LDH concentrations and Hb levels at steady state were comparable for both the standard dosing regimen (LDH: 170 (124-242) U/L; Hb: 11.7 (10.4-13.1) g/dL and the individualized dosing regimen (LDH 174 (125-241) U/L; Hb: 11.7 (10.3-13.0) g/dL. The percentage of patients with LDH normalization (LDH < 226 U/L) at week 16 of treatment were 70.4% for both the standard dosing regimen and individualized dosing regimen. The percentage patient with Hb normalization (Hb >12 g/dL) at week 16 of treatment were 45.6% and 43.6% respectively. The mean yearly maintenance dose costs per patients for the standard dosing regimen and the individualized dosing regimen were US$458,000 and US$420.626, respectively, showing a potential of ~8.2% reduction in yearly drug costs. An intensified dosing interval was necessary in 1.3% of the patients and interval prolongation was possible in 36.7% of the patients. Supplemental Figure S2A-C and S3A-C show the predicted pegcetacoplan trough concentrations, hemoglobin levels and LDH levels at week 16 of treatment for both the approved dosing regimen and the individualized intensified (Figure 2) and prolonged (Figure 3) dosing regimen. For patients with a pegcetacoplan trough concentration of <396 µg/mL and LDH >226 U/L after 7 weeks of treatment, standard dosing will result in pegcetacoplan concentrations of 377.5 (325.3-396.8) µg/mL, Hb levels of 11.6 (10.3-12.7) g/dL and LDH levels of 269 (255-332) U/L at week 16 of treatment. When an intensified dosing regimen of thrice weekly pegcetacoplan was used, pegcetacoplan concentrations increased to 587.0 (504.8-613) µg/mL, Hb levels increased to 11.8 (10.7-13.3) g/dL and LDH were 269 (237-305) U/L at week 16 of treatment.

For patients with a pegcetacoplan trough concentration of >597 µg/mL and LDH <226 U/L after 7 weeks of treatment, standard dosing will result in pegcetacoplan concentrations of 714.5 (653.8-804.3) µg/mL, Hb levels of 11.9 (10.5-13.1) g/dL and LDH levels of 144 (110-181) U/L at week 16 of treatment. When a prolonged dosing regimen was used, pegcetacoplan concentrations decreased to 525 (482-577) µg/mL, Hb levels were 11.6 (10.4-12.8) g/dL and LDH levels were 148 (111-185) U/L at week 16 of treatment.


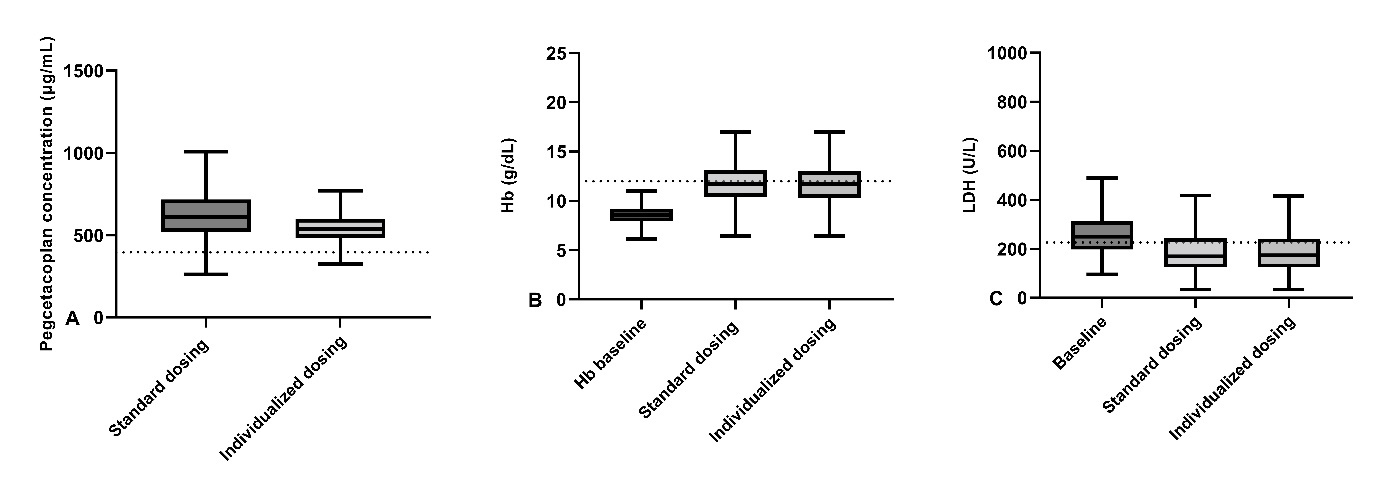


**Figure S1 Tukey box-and-whisker plots for pegcetacoplan trough concentrations (A), Hemoglobin (Hb) levels (B) and lactate dehydrogenase (LDH) (C) at week 16 of treatment for both standard dosing and indiviualized dosing.** The dotted lines represents a pegcetacoplan concentration of 396 µg/ml (EC_90_) (A), a hemoglobin level of 12.0 g/dL (lower limit of normal range for female patients) (B) and an LDH level of 226 U/L (upper limit of normal range) (C).


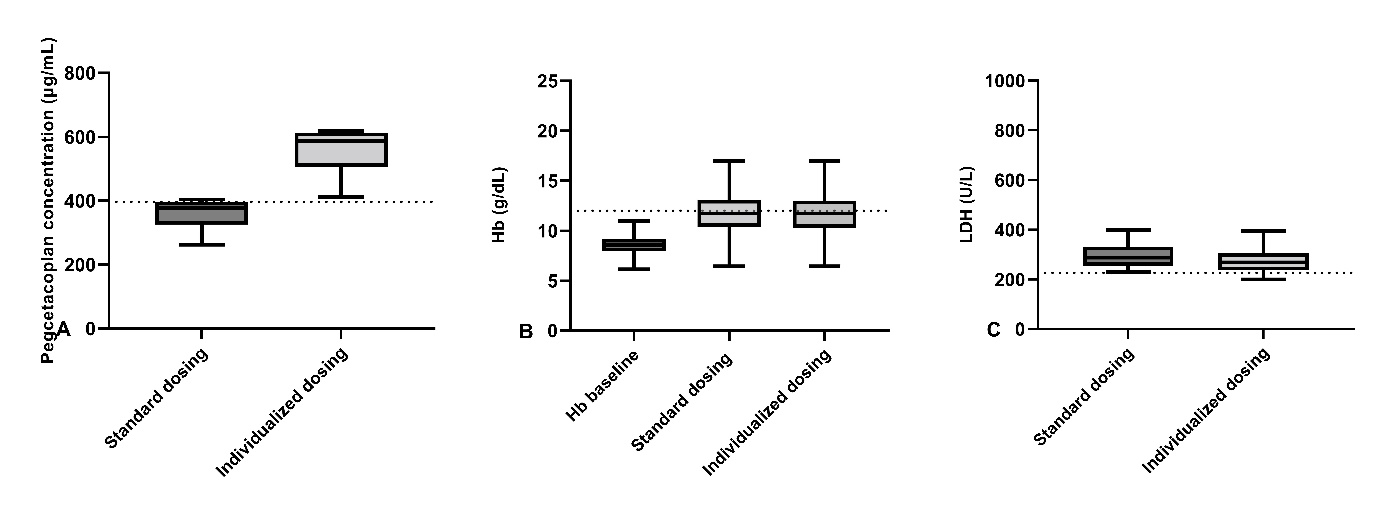


**Figure S2 Tukey box-and-whisker plots for pegcetacoplan trough concentrations (A), Hemoglobin (Hb) levels (B) and lactate dehydrogenase (LDH) (C) at week 16 of treatment for both standard dosing and an intensified individualized dosing strategy for patients with a pegcetacoplan trough concentrations <396 µg/ml after 7 weeks of treatment.** The dotted lines represents a pegcetacoplan concentration of 396 µg/ml (EC_90_) (A), a hemoglobin level of 12.0 g/dL (lower limit of normal range for female patients) (B) and an LDH level of 226 U/L (upper limit of normal range) (C).


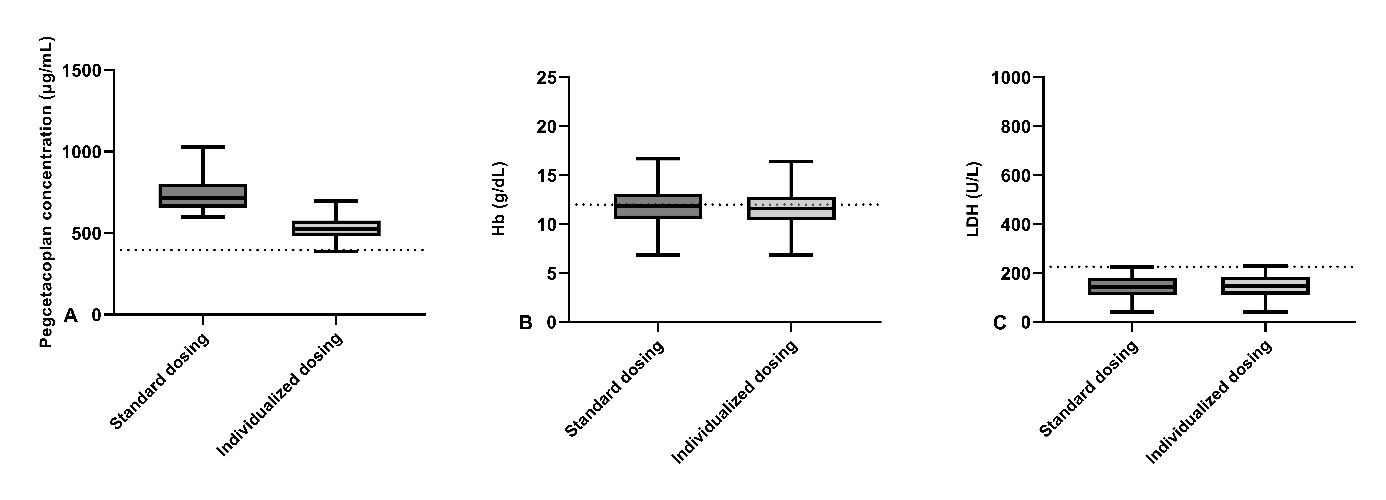


**Figure S3 Tukey box-and-whisker plots for pegcetacoplan trough concentrations (A), Hemoglobin (Hb) levels (B) and lactate dehydrogenase (LDH) (C) at week 16 of treatment for both standard dosing and a prolonged individualized dosing strategy for patients with a pegcetacoplan trough concentrations >597 µg/ml after 7 weeks of treatment.** The dotted lines represents a pegcetacoplan concentration of 396 µg/ml (EC_90_) (A), a hemoglobin level of 12.0 g/dL (lower limit of normal range for female patients) (B) and an LDH level of 226 U/L (upper limit of normal range) (C).

**Nonmem code**

;; 1. Based on: pegcetacoplan data FDA EMA

;; 2. Description: Pegcetacoplan PK/PD sim NO C3 COVARIATE

;; x1. Author: user

$SIZES LVR=32

$PROB PEGCETACOPLAN

$INPUT ID TIME AMT DV CMT MDV EVID WT

$DATA pegcetacoplansimstandaard.csv

$SUBROUTINE ADVAN5

$MODEL

COMP=(SUBCUT) ; S.C. DEPOT COMPARTMENT

COMP=(TRANS) ; TRANSIT ABSORPTION COMPARTMENT

COMP=(CENTRAL) ; CENTRAL OBSERVATION COMPARTMENT

$PK

KA=THETA(1)*EXP(ETA(1))

F1=THETA(2)

CL=THETA(3)*((WT/70)**0.536)*EXP(ETA(2)) ; NO COVARIATE EFFECT OF SERUM C3

V3=THETA(4)*((WT/70)**0.875)*EXP(ETA(3)) ; NO COVARIATE EFFECT OF SERUM C3

S3=V3

K12=KA

K23=KA

K30=CL/V3

$ERROR

IPRED=F

C3=IPRED

Y=IPRED+IPRED*ERR(1)

; PD CODING

; PD HB STEADY STATE

HBBASE=THETA(5)*EXP(ETA(4)) BASELINE HB IN G/dL

CRCL=THETA(6)*EXP(ETA(5)) CRCL 120 ML/MIN FDA REVIEW

HBEMAX=16.129*(HBBASE**(-1.764)) EXTRACTED FROM FDA REVIEW TEXT BY FITTING POWER TRENDLINE: HB 6.2--> EMAX 0.611, HB 8.59 --> EMAX 0.386, HB 10.6--> EMAX 0.251.

CRCLEMAX=(0.0014*CRCL)+0.8107 EXTRACTED FROM FDA REVIEW TEXT BY FITTING LINEAR TRENDLINE: CRCL 50--> EMAX 0.872, CRCL 120--> EMAX 1, CRCL 221 --> EMAX 1.12.

EMAXHB=(HBEMAX*CRCLEMAX)*EXP(ETA(6)) EMAX WITH IIV

EC50HB=THETA(7)*EXP(ETA(7)) EC50 IN UG/ML OR MG/L --> EC90=400 UG/ML

HILLHB=THETA(8) OR GAMMA

HB=(HBBASE*(1+((EMAXHB*(C3**HILLHB))/((EC50HB**HILLHB)+(C3**HILLHB)))))+ETA(10) ; HB

; --- PD LDH AT STEADY STATE WITHOUT ECULIZUMAB AT BASELINE

LDHBASE=THETA(9)*EXP(ETA(8)) 2117 IN ABSENCE OF ECULIZUMAB AND 248 IN PRESENCE OF ECULIZUMAB

EMAXLDH=THETA(10) 0.908 IN ABSENCE OF ECULIZUMAB AND -0.325 IN PRESENCE OF ECULIZUMAB

EC50LDH=THETA(11)*EXP(ETA(9)) 173 MG/L IN ABSENCE OF ECULIZUMAB AND 250 IN PRESENCE OF ECULIZUMAB

HILLLDH=THETA(12) OR GAMMA

LDH=(LDHBASE*(1+((EMAXLDH*(C3**HILLLDH))/((EC50LDH**HILLLDH)+(C3**HILLLDH))))) * EXP(ETA(11)) ; LDH

; --- PD LDH AT STEADY STATE WITH ECULIZUMAB AT BASELINE

LDHBASEEC=248*EXP(ETA(8)) 2117 IN ABSENCE OF ECULIZUMAB AND 248 IN PRESENCE OF ECULIZUMAB

EMAXLDHEC=-0.325 0.908 IN ABSENCE OF ECULIZUMAB AND -0.325 IN PRESENCE OF ECULIZUMAB

EC50LDHEC=250*EXP(ETA(9)) 173 MG/L IN ABSENCE OF ECULIZUMAB AND 250 IN PRESENCE OF ECULIZUMAB

LDHEC=(LDHBASEEC*(1+((EMAXLDHEC*(C3**HILLLDH))/((EC50LDHEC**HILLLDH)+(C3**HILLLDH))))) * EXP(ETA(11)) ; LDH

$THETA

; --- PK

(0, 0.0394) 1 KA PER HOUR TABLE 3 EMA ASSESSMENT REPORT

(0, 0.766) 2 F1 SOLUTION FORMULATION TABLE 3 EMA REPORT

(0, 0.015) 3 CL L/H PNH FROM EMA SPC

(0, 3.9) 4 L PNH VOLUME FROM EMA SPC

; --- PD HB

(0, 8.59) 5 HBBASE G/DL

(0, 120) 6 CRCL ML/MIN

(0, 272) 7 EC50 HB

(0, 5.85) 8 HILL HB

; --- PD LDH

(0, 2117) 9 LDH BASELINE IU/L

(-10, -0.908) 10 EMAX IN ABSENCE OF ECULIZUMAB AT BASELINE

(0, 173) 11 EC50 IN MG/L IN ABSENCE OF ECULIZUMAB

(0, 3.84) 12 HILL LDH

$OMEGA

0.215 1 IIV KA

$OMEGA BLOCK(2)

0.0380 2 IIV CL

0.0268 0.0462 2~3 CORRELATION AND 3 IIV V

$OMEGA

0.0121 4 IIV HB BASE

0.09 5 IIV CRCL 30% ASSUMPTION

0.210 6 IIV EMAX HB

0.162 7 IIV EC50 HB

0.114 8 IIV LDH BASE

0.158 9 IIV IIV EC 50 LDH

1.02 10 ADD ERR HB

0.120 11 PROP ERR LDH

$SIGMA

0.0128 ; PROP ERR PNH PHASE 3

$SIM ONLYSIM SUBPROBLEMS=1 (2252) (74292)
